# Experimental study of learning support through examples in mathematical problem posing

**DOI:** 10.1007/s41039-015-0001-5

**Published:** 2015-06-23

**Authors:** Kazuaki Kojima, Kazuhisa Miwa, Tatsunori Matsui

**Affiliations:** 1 Learning Technology Laboratory, Teikyo University, 1-1 Toyosatodai, Utsunomiya, 320-8551 Japan; 2 Graduate School of Information Science, Nagoya University, Furocho, Chikusaku, Nagoya, 464-8601 Japan; 3 Faculty of Human Sciences, Waseda University, 2-579-15 Mikajima, Tokorozawa, 359-1164 Japan

**Keywords:** Production task, Problem posing, Learning from examples, Mathematical learning

## Abstract

When using mathematics to solve problems in everyday life, problem solvers must recognize and formulate problems by themselves because structured problems are not provided. Therefore, in general education, fostering learner problem posing is an important task. Because novice learners have difficulty in composing mathematical structures (*solutions*) in problem posing, learning support to improve the composition of solutions is required. Although learning by solving examples is adopted in general education, it may not be sufficiently effective in fostering learner problem posing because cognitive skills differ between problem solving and problem posing. This study discusses and experimentally investigates the effects of learning from examples on composing solutions when problem posing. We studied three learning activities: learning by solving an example, learning by reproducing an example, and learning by evaluating an example. In our experiment, undergraduates were asked to pose their own new, unique problems from a base problem initially presented after the students learned an example by solving, reproducing, or evaluating it. The example allowed the undergraduates to gain ideas for composing a novel solution. The results indicated that learning by reproducing the example was the most effective in fostering the composition of solutions.

## Background

In addition to solving problems provided by a teacher or textbook, problem posing, by which learners create problems, has also been identified as an important activity in mathematics education. In fact, some mathematicians and mathematics educators have pointed out that problem posing lies at the heart of mathematical activity (English 1997; Polya 1945; Silver 1994). Problem posing is a necessary skill for problem solving in everyday life. Because structured problems are not provided when using mathematics in everyday life, problem solvers must recognize and formulate problems by themselves (Ishida and Inoue 1983; Singer and Voica 2013). Therefore, it is an important task in general education to foster learner problem posing. Several studies have addressed this issue in terms of a learning activity to improve problem solving, despite insufficiently addressing the skill of learner problem posing itself.

The problem-posing research has empirically confirmed that novice learners succeeded in posing new problems based on mathematical structures provided in formulae or equations, whereas they had difficulty in composing novel mathematical structures on their own (Christou et al. 2005; Kojima et al. 2010). Because problem posing in everyday life is performed under various constraints with different materials, fostering the skill to pose diverse problems appropriately is highly desirable. In human problem solving, two attributes of problems are recognized as crucial: one is surface features such as contextual settings in problem texts (e.g., purchase of goods or transfer by vehicles) and the other is structural features such as mathematical structures (Gentner 1983; Forbus et al. 1995; Holyoak and Thagard 1996). We refer to these two attributes as *situations* and *solutions*. Studies in cognitive science have demonstrated that novice learners are strongly influenced by situations in problem solving and often fail in understanding solutions and adapting them to problem solving (e.g., Novick 1988; Reed et al. 1985; Ross 1987). Similarly in problem posing, composing novel solutions is more difficult than generating new situations. Therefore, learning support is required to improve the composition of solutions by novice learners. Here, composition of solutions is a process in which a problem poser generates mathematical relationships and then forms equations and stories in texts along with the relationships. Problem posing in everyday life must require composition of solutions from information given to or generated by the poser. Our study addressed improvement of solution composition as prerequisite for fostering problem-posing skill.

To support novice learners, using examples is efficient and effective. Examples are indispensable for learning in any domain, including mathematics. In general mathematics education, procedures for solving problems are initially taught using examples. Cognitive science studies have also argued how to foster transfer of a solution learned in an example to problem solving (e.g., Gick and Holyoak 1983; Novick and Holyoak 1991). However, the general method of learning from examples in problem solving may not be sufficiently effective in problem posing because cognitive skills in problem solving and problem posing are different. We refer to the former task as a comprehension task and the latter as a production task. In fact, some researchers report that learning tasks such as comprehension and production have no mutual influence (Singley and Anderson 1989). Accordingly, to improve composition of solutions in problem posing, learning examples using production tasks may be more effective than those using comprehension tasks.

To provide a basis for computational support that uses examples in problem posing, this study discussed and experimentally investigated the effects of activities for learning from an example on composing solutions in problem posing. In our experiment, undergraduates were asked to pose their own new, unique problems from a base problem initially presented after they had learned an example. We compared three activities for learning from an example adopted in general mathematical education or computational support systems for problem posing. In the next session, we discussed problem posing and the activities of learning from problem-posing examples.

## Theoretical background

### Relationships and differences between problem solving and problem posing

Although problem solving and problem posing differ, they are not entirely different cognitive activities but are closely related. Several researchers have experimentally confirmed that problem-solving ability and problem-posing performance are correlated and that problem posing positively influences problem solving (Bernardo 2001; Ellerton 1986; Nikata and Shimada 2005; Silver and Cai 1996). Problem posing offers many benefits: For example, it enhances problem-solving ability and the grasp of mathematical concepts, generates diverse and flexible thinking, alerts both teachers and learners to misunderstandings, and improves learners’ attitudes and confidence in mathematics (English 1998; Silver 1994). Although problem posing is rarely adopted in general education owing to certain constraints in practical classrooms, it is as critical a skill as problem solving.

Problem solving and problem posing differ, of course, in the features and formats of their tasks. Problem solving is a comprehension task, by which a learner extracts a mathematical structure from given information and reaches a correct answer. In contrast, problem posing is a production task that requires generation of information and its synthesis. Learners show difficulty in problem posing even if they can easily solve the problems. Akay and Boz (2009) asked prospective science teachers to respond to questionnaires about problem posing after participation in a course oriented to mathematical problem posing. The prospective teachers responded that problem posing was difficult because of its nature (e.g., not knowing the steps of problem posing), their abilities (not being creative), or lack of mathematical knowledge (having difficulties understanding abstractions) although they were not novices but had been trained as teachers.

Base (A_1_): I bought some 60-yen oranges and 120-yen apples for 1020 yen. The total number of oranges and apples was 12. How many oranges and apples did I buy?

Solution:

Let *x* denote the number of oranges and *y* denote the number of apples.

*x* + *y* = 12

60*x* + 120*y* = 1020

According to the equations above, *x* = 7 and *y* = 5.

**Fig. 1:**
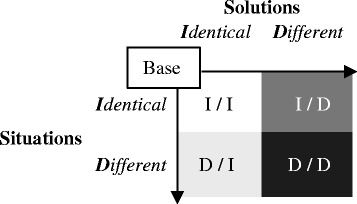
Categories for evaluating posed problems

**Fig. 2:**
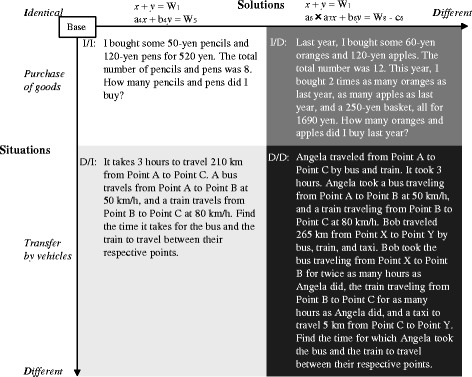
Examples of posed problems in each category

We investigated problems posed by novices to understand the difficulties they encounter in problem posing (Kojima et al. 2010). Undergraduates were asked to generate new problems from problems initially presented as bases. The bases were simple word problems easily solved by equations. The undergraduates were then encouraged to generate problems as varied and unique as possible. The variety of problems they posed was evaluated according to the four categories shown in Fig. 1, indicating similarities in the situations and solutions between each of their problems and the bases. Category I/I indicates problems that are almost the same as the bases, D/I indicates problems generated by altering the situations of the bases, I/D indicates problems generated by altering the solutions, and D/D indicates problems generated by combining alterations in both situations and solutions. Figure 2 presents examples of problems posed in each category that were solved by simultaneous equations. The results confirmed that the undergraduates posed many problems in categories I/I and D/I and few problems in I/D. They also revealed that D/I problems with situations different from the bases were appropriately composed. On the other hand, problems in I/D and D/D, where solutions differed from the bases, were relatively simple and inappropriate. Although the bases were elementary problems, many of the posed problems were simpler than the bases. These results indicate that the novices could generate novel situations, but failed to create new solutions in problem posing; thus, even if they can easily solve problems, undergraduates have difficulty in posing new problems. Therefore, because problem posing is more difficult than problem solving, it requires additional support.

Computational support of learning by problem posing has already been developed in various domains (Barak and Rafaeli 2004; Hirashima et al. 2007; Hirashima et al. 2010; Hirai et al. 2009; Takagi and Teshigawara 2006; Yu et al. 2005). However, such computational support focuses mainly on improving performance of comprehension tasks through problem posing, such as understanding domain knowledge or procedures in problem solving. Some studies empirically analyzed problems posed by learners (e.g., Cankoy 2014; English 1998; Leung 1997; Yu and Wu 2013); however, these studies have not addressed learning from examples in problem posing.

### The effects of examples in problem posing

The research field of Intelligent Tutoring Systems/Artificial Intelligence in Education has long addressed learning from examples. Interactive scaffolding that enhances learning from examples has been implemented, and its effects have been discussed (e.g., Conati and VanLehn 2000; Koedinger and Aleven 2007; Schwonke et al. 2009; McLaren and Isotani 2011). However, the central issue in such research is basically limited to problem solving and does not include problem posing.

Even so, some studies have addressed learning from examples in problem posing. Hsiao et al. (2013) experimentally confirmed the effect of seeing worked examples on problems posed by undergraduates in the business mathematics domain. The undergraduates posed problems with a web-based learning management system in three homework exercises after lecture classes. In each exercise, half of the students were provided two problems as examples solved through concepts or formulae learned in the lecture classes. The results demonstrated the effects of the examples: undergraduates who provided examples posed fewer problems not oriented to what they had learned in the lecture classes than those who provided no examples. Hsiao et al. also examined problems posed by undergraduates in terms of complexities. However, the examples’ effects on the problems’ complexities were limited—the examples did not expand the average complexity of each posed problem. Because Hsiao et al. provided each as a worked example, the undergraduates must have read only its solution, indicating that they learned the example through comprehension tasks.

We implemented a support system to facilitate learners’ posing of diverse problems by using examples (Kojima and Miwa 2008). In the system, learners engage in the same task as the one described above (Kojima et al. 2010). They pose new problems and input the texts and equations of their solutions into the system. The system automatically understands the situations and solutions in the problems and evaluates their variety. It can also present learners with problems as examples to provide hints for idea generation. The variety of learners’ problems is evaluated, and the presentation of examples is controlled on the four-category basis shown in Fig. 1. Experimental evaluations of the system confirmed that to some extent, it could facilitate learners’ posing of diverse problems. The number of problems posed in the I/I category decreased and those in D/I and D/D increased after the learners had posed problems with the system, and the system showed them various examples belonging to D/I and I/D. However, the presentation of examples did not increase the number of problems in I/D. The lack of problem posing in I/D was consistent with the results obtained by Kojima et al. (2010).

Although the system presents examples to learners and prompts them to compare the base with their posed problems, it does not give any instructions on how to learn from the examples. The examples are merely shown to the learners. We have not examined how the learners actually learned from the presented examples: the learners may have simply read the presented examples. In other words, the learners may have understood the examples through performing a comprehension task. The comprehension of examples may have helped in generating various situations; however, it may not have necessarily facilitated understanding of the solution structures. For learners to adequately study the solutions from examples and transfer that knowledge to their problem posing, further support must be introduced. Because problem posing is a production task, it effectively allows a learner to examine each example through a productive activity.

### Learning activities of examples in problem posing

#### Learning by solving examples

Solving examples and understanding the solution is of course one of the most popular activities in mathematical learning. As mentioned above, however, learning by solving may not be effective in improving the composition of solutions in learner problem posing because problem solving differs from problem posing.

#### Learning by reproducing examples

We designed a method of learning from examples through imitation, a learning activity adopted in productive task domains (Kojima et al. 2013). Imitation—the method by which learners reproduce existing example works—has long been adopted as a major learning activity in the domains of creative generation, such as art and music. The relationship between imitation and creation has been consistently noted in such domains and the effects of imitation have been documented. For example, Ishibashi and Okada (2006) argue that imitating examples can prompt imitators’ understanding of examples and their conceptual background; imitation facilitates a creative performance by imitators. In their experiment, subjects were engaged in an artistic drawing task before and after they created copies of a presented example. Results showed that the subjects deeply understood the example through its imitation, and understanding the example then elicited understanding of the subjects’ own expressions.

Based on this insight, we implemented a system for learning by reproducing examples in problem posing (Kojima et al. 2013) as an enhancement of the system previously described (Kojima and Miwa 2008). Learning by reproduction of an example allows learners to understand the ideas used in formulating the example from the viewpoint of the poser.

**Fig. 3:**
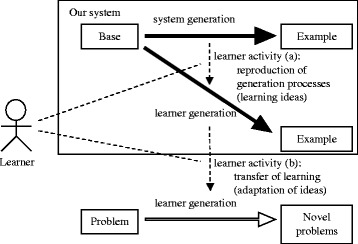
Basic framework of learning by reproducing examples

Figure 3 indicates the basic framework for learning by reproducing examples. In learning with the system, a learner is required to pose new problems from an initially given base. The learner is also presented with problems as examples, each generated by altering the base. When a learner studies an example generated from the base, the system hides the example itself and shows its generation process information to indicate how it was generated (bold black arrows in Fig. 3). Generation process information also includes sufficient information to reproduce the example. The learner generates a problem identical to the example by reproducing alteration of the base as indicated in generation process information (Fig. 3(a)). This prevents the learner from merely duplicating the characters and symbols that compose the text and solution of the example. From a poser’s viewpoint, this learning activity can facilitate understanding of the essential ideas used to generate the example, particularly those for composing a solution. The learner then transfers what is learned through reproduction into the posing of new problems (Fig. 3(b)).

We experimentally verified that learning by reproducing an example facilitated problem posing through directly adopting ideas used in the example’s generation (Kojima et al. 2013). However, we have not yet confirmed whether such learning can foster composition of solutions in the learner’s own problem posing.

#### Learning by evaluating examples

Some computational systems for supporting learning by problem posing (e.g., Barak and Rafaeli 2004; Hirai et al. 2009; Takagi and Teshigawara, 2006; Yu et al. 2005) adopt problem evaluation among learners as an activity in addition to problem posing. Experiments have shown that learning through such activities improves learning performance as well as the quality of learner problems. These studies basically designed the systems from the viewpoint of collaborative learning and focused on improving understanding of domain knowledge through problem posing. Although it is empirically confirmed that evaluations of problems posed by learners had predictive effects on the problems’ qualities (Yu and Wu 2013), these studies have not immediately produced evidence about the cognitive impacts of a learner evaluating activity on problem posing by the learners themselves.

Evaluation is a process involved in the creative generation of ideas or products. The importance of evaluative skills in creativity has been documented (Runco and Chand 1994). Furthermore, the effects of evaluating examples on the evaluator’s idea generation have been empirically demonstrated. Lonergan et al. (2004) experimentally observed that evaluation of examples according to certain standards improved the originality and feasibility of ideas generated by the evaluators, depending on the qualities of the examples and standards. Therefore, evaluation of existing ideas or products can be regarded as a production task because evaluation is a cognitive activity that can contribute to creative generation.

According to the above-mentioned studies, we experimentally investigated the effects of learning from an example on solution composition for problem posing. In the investigation, we studied the learning activities of reproducing and evaluating an example. To examine differences between comprehension and production tasks, we also studied the effects of learning by solving the same example. Because novice learners pose few such problems as examples, the investigation used an I/D problem as an example of a problem having a solution more complex than the base. As mentioned above, it is important to foster posing such problems because composing novel solutions is necessary but difficult, whereas generation of new situations is easy.

## Methods

### Procedures and materials

Undergraduates participated in the experimental investigation conducted in three classes of a cognitive science lecture from 2010 to 2012. The undergraduates were first given a learning task in the domain of word problems solved with simultaneous equations. They were told that the learning task’s purpose was to instruct them how to pose a novel problem from a base. The base in the learning task was the problem A_1_ (see Fig. 2). The undergraduates learned the following problem, A_2_, as an example of output in the domain of A_1_.

A_2_: Last year, I bought some 40-yen pencils and 110-yen pens. The total number was 13. This year, I bought 2 times as many pencils as last year, as many pens as last year, and a 300-yen pen case for 1430 yen. How many pencils and pens did I buy last year?

Solution.

Let *x* denote the number of pencils and *y* denote the number of pens.

*x* + *y* = 13

40 × 2*x* + 110*y* = 1430–300

According to the equations above, *x* = 10 and *y* = 3.

The solution of A_2_ was composed by an alteration that added two parameters and operations to A_1_. Thus, this process can hint at composing complex solutions in problem posing by the undergraduates. A_2_ is an I/D problem more complex than the base and difficult to pose for novice learners. The undergraduates had to learn the example in 15 min.

The learning task was followed by a problem-posing task, in which the undergraduates were asked to pose their own problems in the domain of word problems solved with unitary equations. The base in the problem-posing task was the following problem B.

B: I want to buy some boxes of cookies. If I buy 110-yen boxes of cookies, then I have 50 yen left. If I buy 120-yen boxes of chocolate cookies, then I need 20 yen more. How many boxes do I want?

Solution.

Let *x* denote the number of boxes.

110*x* + 50 = 120*x* − 20

According to the above equation, *x* = 7.

Prior to the problem-posing task, the undergraduates were instructed to pose as many diverse and unique problems as possible in 20 min.

### Condition groups

Undergraduates in the same school of the same university participated as one of the three condition groups each year. Because it is an interdisciplinary school, the background of the undergraduates varied but no one majored in mathematics. All of the undergraduates had trained to solve problems in the domain of word problems solved with linear equations in middle and high school education.

In the 2010 class, undergraduates were provided sheets of paper on which the text and solution of A_1_ and the text of A_2_ were printed. They were asked to solve A_2_ and write the answer on the sheet. The undergraduates were hence referred to as the solving group.

In 2011, undergraduates were first presented A_1_ and A_2_ on a screen. After A_2_ had been removed from the screen, they were provided printed sheets with A_1_ and generation process information indicating how to compose A_2_ from A_1_. The generation process information had been created by the system mentioned above (Kojima et al. 2013). The undergraduates were asked to reproduce, according to the information, the same problem as A_2_. They were also told that their problems’ texts did not need to be identical with the example as long as the problems could be solved by a solution identical to the example. We refer to the undergraduates as the reproduction group. Appendix 1 shows the information presented to this group.

In 2012, undergraduates were provided sheets on which A_1_ and A_2_ were printed. They were asked to evaluate A_2_ with a view toward originality and feasibility as a mathematical problem by using a 5-point scale and to describe the reasons for the evaluations. These viewpoints are generally used in researching creative thinking (e.g., Finke et al. 1996). We refer to these undergraduates as the evaluation group.

### Analysis

In fact, transfer of the example enabled posing I/D problems whose solutions were more complex than the base by altering the solution of the base. To verify the effect, we examined the following research questions:

RQ1: Do the undergraduates pose I/D problems after learning the example?

RQ2: Do the undergraduates learn how to compose solutions by altering the example and transferring it to their problem posing?

RQ3: After they learn the example, are the undergraduates fostered to compose solutions more complex than the base?

Problems posed by the undergraduates were analyzed in terms of variety, strategies to alter solutions, and complexities of solutions. To examine RQ1, the variety of each problem was evaluated on the basis of the four categories shown in Fig. 1. The example A_2_ is a problem in category I/D.

To examine RQ2, strategies to alter solutions of problems posed by the undergraduates were evaluated by comparing each problem’s solution structure with that of the base. The undergraduates’ problems were classified into *not altered*, *partially altered* (adding/removing operations to/from the solution of the base), or *overall altered* (composing a solution entirely different from the base). A_2_ was posed with partially altered.

To examine RQ3, the complexities of the undergraduates’ problems were estimated by comparing the numbers of operations required to reach the answers with the number required for the base. The number of operations in the base is three. Only the complexities of I/D and D/D problems were analyzed because the structure of solutions in I/I and D/I problems are always equal to the base.

In the study previously described (Kojima et al. 2010), we acquired problems posed by undergraduates in the same task without any learning through example in another class of the cognitive science lecture in 2009. The effects of learning with the example were verified through a comparison of the solving, reproduction, or evaluation groups in this investigation as *experimental groups* with those of the previous study as a *control group*. The procedures and material of the problem-posing task in the control group were the same as those described in the “Procedures and materials” section. For the comparison, this study used the same problem-posing task.

In the reproduction group, some undergraduates did not reproduce A_2_ and instead posed problems that were slightly different from A_2_ (e.g., changing parameters or operations in A_2_); some others did not complete reproduction in the learning task. Such undergraduates were excluded from the analysis. Some others in the reproduction group failed to reproduce A_2_. Although they wrote the same solution as A_2_, their problem texts were contradictory to the solution. Therefore, the data of those who failed in the learning task (*reproduction-f group*) were separately described from those who succeeded (*reproduction-s group*). Appendix 2 shows an example of a contradictory problem posed by the reproduction-f group.

## Results

In the solving group, 62 undergraduates participated; in the reproduction group, 132; and in the evaluation group, 25. In the reproduction group, 44 did not reproduce A_2_, and 8 did not complete reproduction. In the others, 52 were in the reproduction-s group, and 28 were in the reproduction-f group. Undergraduates in the solving, reproduction-s, reproduction-f, and evaluation groups posed 372 problems in the problem-posing task, 68 of which were excluded because they were in domains other than the base (e.g., solved with inequalities) or unsolvable due to insufficient or contradictory constraints. Because the undergraduates were instructed to pose problems in the domain of the base, posing any problems in other domains was a violation of the instruction. In case of unsolvable problems, solutions described by undergraduates were inconsistent with problem text that they described. Thus, problems that the undergraduates tried to pose were unclear. Appendix 3 shows some examples of problems posed in the experimental groups. In the control group, 76 undergraduates participated. They posed 146 problems and 29 were excluded in the same manner.

### Variety

**Fig. 4:**
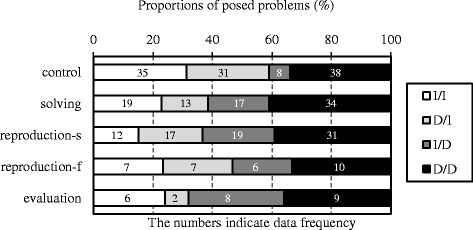
Proportions of posed problems in each category

Figure 4 indicates the proportions of posed problems in each category, and Table 1 indicates differences in the numbers between the control and each of the experimental groups. As mentioned above, the control group posed few I/D problems. The experimental groups posed more I/D problems than the control group. We compared the control group with the solving group using the chi-square test; the result indicated a significant difference between the solving and control groups (*χ* ^2^(3) = 11.51, *p* < .01). Furthermore, the results of residual analysis indicated that the number of D/I problems was significantly high in the control group but significantly low in the solving group. The number of I/D problems was significantly high in the solving group but significantly low in the control group. Similarly, a significant difference existed between the reproduction-s and control groups (*χ* ^2^(3) = 15.26, *p* < .01). The number of I/I problems was significantly high in the control group but significantly low in the reproduction-s group. The number of I/D problems was significantly high in the reproduction-s group but significantly low in the control group. There was also a significant difference between the evaluation and control groups (*χ* ^2^(3) = 14.48, *p* < .01). The number of D/I problems was significantly high in the control group but significantly low in the evaluation group, whereas the number of I/D problems was significantly high in the evaluation group but significantly low in the control group. There was no difference between the reproduction-f and control groups (*χ* ^2^(3) = 4.64, n.s.).

**Table 1:** The numbers of posed problems in each category

Groups	I/I	D/I	I/D	D/D
Control	35	31*	8**	38
Solving	19	13*	17**	34
Control	35*	31	8**	38
Reproduction-s	12*	17	19**	31
Control	35	31	8	38
Reproduction-f	7	7	6	10
Control	35	31*	8**	38
Evaluation	6	2*	8**	9

*p < .05; **p < .01

### Solution-altering strategies

**Fig. 5:**
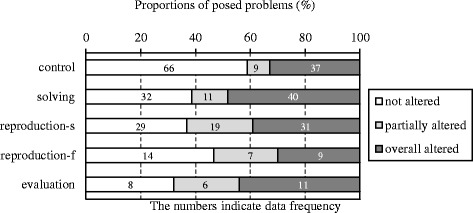
Proportions of posed problems with each solution-altering strategy

Figure 5 indicates the proportions of posed problems composed with each solution-altering strategy in each group, and Table 2 indicates differences in the numbers between the control and each of the experimental groups. The chi-square test indicated a significant difference between the solving and control groups (*χ* ^2^(2) = 7.98, *p* < .05). Furthermore, the results of residual analysis indicated that the number of not altered problems was significantly high in the control group but significantly low in the solving group, whereas the number of fully altered problems was significantly high in the solving group but significantly low in the control group. Similarly, there was a significant difference between the reproduction-s and control groups (*χ* ^2^(2) = 13.20, *p* < .01). The results of residual analysis indicated that the number of not altered problems was significantly high in the control group but significantly low in the reproduction-s group. The number of partially altered problems was significantly high in the reproduction-s group but significantly low in the control group. There was also a significant difference between the evaluation and control groups (*χ* ^2^(2) = 8.20, *p* < .05). The results of residual analysis indicated that the number of not altered problems was significantly high in the control group but significantly low in the evaluation group, whereas the number of partially altered problems was significantly high in the evaluation group but significantly low in the control group. There was a moderate but significant difference between the reproduction-f and control groups (*χ* ^2^(2) = 5.61, *p* < .10). The results of residual analysis indicated that the number of partially altered problems was significantly high in the reproduction-f group but significantly low in the control group.

**Table 2:** The numbers of posed problems with each solution-altering strategy

Groups	Not altered	Partially altered	Overall altered
Control	66**	9	37*
Solving	32**	11	40*
Control	66**	9**	37
Reproduction-s	29**	19**	31
Control	66	9*	37
Reproduction-f	14	7*	9
Control	66*	9*	37
Evaluation	8*	6*	11

*p < .05; **p < .01

### Complexities

**Fig. 6:**
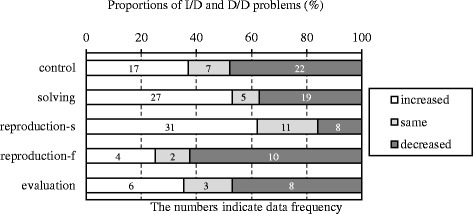
Proportions of altered problems whose operations increased or decreased

Figure 6 indicates the proportions of I/D and D/D problems whose number of operations increased or decreased from the base, and Table 3 indicates differences in the numbers between the control and each of the experimental groups. In half of the I/D and D/D problems posed by the control group, the number of operations decreased from the base, implying that half of the I/D and D/D problems were simpler than the base. The number of such simple problems was smaller only in the reproduction-s group. We compared the control group with the solving, reproduction-s, reproduction-f, and evaluation groups using the chi-square test, with the results indicating a significant difference between the reproduction-s and control groups (*χ* ^2^(2) = 11.36, *p* < .01). Furthermore, the results of residual analysis indicated that the number of decrease was significantly high in the control group but significantly low in the reproduction-s group. The number of increase was significantly high in the reproduction-s group but significantly low in the control group. There was no difference between the solving and control groups (*χ* ^2^(2) = 2.58, n.s.), the reproduction-f and control groups (*χ* ^2^(2) = 1.06, n.s.), or the evaluation and control groups (*χ* ^2^(2) = 0.06, n.s.).

**Table 3:** The numbers of altered problems whose operations increased or decreased

Groups	Increased	Same	Decreased
Control	17	7	22
Solving	27	5	19
Control	17*	7	22**
Reproduction-s	31*	11	8**
Control	17	7	22
Reproduction-f	4	2	10
Control	17	7	22
Evaluation	6	3	8

*p < .05; **p < .01

## Discussion and conclusion

The results presented above indicate that the experimental groups posed more I/D problems than the control group, indicating that the example facilitated posing I/D problems regardless of the learning activities. Thus, RQ1 was verified in all of the experimental groups.

On the other hand, there was a difference among the experimental groups in the solution-altering strategies. Overall altered problems posed increased in the solving group, whereas partially altered problems posed increased in the production-s, production-f, and evaluation groups. The latter three groups adapted ideas used in the example because it was composed by altering the base (partially altered). The solving group learned the example through a comprehension task, whereas the reproduction-s, reproduction-f, and evaluation groups did so through a production task. Therefore, RQ2 was verified in the reproduction-s, reproduction-f, and evaluation groups, demonstrating that learning the example through a production task facilitated its transfer to the undergraduates’ problem posing.

The results shown in Figs. 4 and 5 confirm that learning the example increased production of problems whose solutions differed from the base. As pointed out in the introduction, novices find it difficult to compose novel solutions when problem posing. The experimental groups posed problems with novel solutions in some senses, even though only the reproduction-s group posed many problems more complex than the base. The undergraduates could learn how to formulate more complex solutions by adding operations. However, such problem posing was performed only by those who had succeeded in reproducing the example. According to this, RQ3 was verified only in the reproduction-s group. Therefore, the answers to the research questions were as follows:

RQ1: Do the undergraduates pose I/D problems after learning the example? Yes, the example increased I/D problems.

RQ2: Do the undergraduates learn how to compose solutions by altering the example and transferring it to their problem posing? Partially, yes. Those who had learned the example through a production task transferred it.

RQ3: After they learn the example, are the undergraduates fostered to compose solutions more complex than the base? Partially, yes. Only those who succeeded in reproducing the example produced solutions more complex than the base.

The results indicate that learning by solving an example can increase I/D problems. In the previous study (Kojima and Miwa 2008), learners just viewed the examples. Therefore, involving learners with an example is effective to some extent in problem posing. On the other hand, such involvement is not sufficiently effective in fostering the composition of solutions.

The results also prove that in problem posing, learning an example through a production task is effective. The results also confirm that learning by reproducing an example is more effective in terms of a learning activity in a production task. However, this activity also involves difficulty. Although no one in the solving group failed in the learning task, the reproduction-f group did fail. Obviously, the example must be quite easy for undergraduates to solve. Although learning by reproduction is effective, it significantly challenges learners. Therefore, further supportive intervention must be introduced in learning from an example through a production task.

The reproduction-s and the evaluation groups both adapted the example to the problem-posing task. The evaluation group posed many I/D problems, as well as partially altered problems. However, like the control group, the evaluation group posed many I/D and D/D problems that were simpler than the base. Although this group evaluated the example as to its originality and feasibility, alternative viewpoints might be needed to improve an example’s effects. Furthermore, to enhance the effects of evaluation, presenting a nasty problem as an example is one alternative. A learner may devise a good idea through evaluating such an example and find how to improve the example. Further study is needed to thoroughly examine this point.

The results of the solving and reproduction-s groups were consistent with the report by Singley and Anderson (1989). They experimentally confirmed that there was little transfer from training of evaluating LISP code to generating LISP code and vice versa. In the same way, this study confirmed that solving the given example did not effectively transfer to posing new problems. On the other hand, reproducing the example fostered problem posing while transferring ideas used in the example. It indicates that experience to follow processes of generating the example was required for learning in problem posing. Therefore, the effects of learning the example were insufficient in the evaluation group because of the absence of such experience to follow generation processes.

This study has limitation in terms of influence of individual aspects of the undergraduates on problem posing. Mathematical abilities such as reasoning skills (Ellerton 1986) and some other variables such as self-efficacy in mathematics and attitudes toward mathematics (Akay and Boz 2010) can positively influence on behaviors and products in problem posing. This study has not addressed these aspects. We have to further study the influence of these aspects on the effects of learning examples in problem posing.

## Appendices

### Appendix 1

The example was composed by altering the base in the ways described below. According to these, make a problem identical to the example. It is unnecessary to exactly reproduce the text of the example as long as your problem is solved with the same solution.

#### *x* and *y*

Objects are altered to “pencils” and “pens”

*x*: pencils

*y*: pens

Answers: *x* = 10, *y* = 3 (how many)

#### Numeric parameters in the text

Two parameters are added

Parameters: (total) 13, pen 110 yen, pencil 40 yen, pencil 2 times, (total) 1430 yen, pen case 300 yen

Third object (pen case and 300 yen) is added

#### Solution

Altered from the base

[*x* pencils] + [*y* pens] = [total 13]

[*1] × [*x* pencils] + [110 yen pen] × [*y* pens] = [*2]

*1 Operation [40 yen pencil] × [2 times pencils] is added

*2 Operation [total 1430 yen] − [pen case 300 yen] is added

#### Problem text

Keywords: last year, pencils, pens, total, buy, this year, the number, pen case

### Appendix 2

#### An example posed by the reproduction-f group in the learning task

Last year, I bought 40-yen pencils and 110-yen pens and a 300-yen pencil box for 1430 yen^a^. The total number of pencils and pens was 13. This year, I bought 2 times as many pencils as last year and as many pens as last year. The total number this year was also 13^b^. Their sum was the same as last year, excluding the pencil box^c^. How many pencils and pens did I buy this year?

Let *x* denote the number of oranges and *y* denote the number of apples.

*x* + *y* = 12

60*x* + 120*y* = 1020

According to the equations above, *x* = 7 and *y* = 5.

^a^Not last year, but this year

^b^Not 13 this year

^c^The sums this year differed from last year

### Appendix 3

#### Examples of problems posed in the problem-posing task

##### D/I problem posed in the solving group

A teacher planned to divide students into groups of equal numbers of students. If 5 students were assigned to each group, then 2 students were left. If 6 students were assigned to each group, then 4 additional students were needed. How many groups did the teacher want to make?

Solution.

Let *x* denote the number of groups.

5*x* + 2 = 6*x* − 4

*x* = 6.

##### I/D problems posed in the production-s group

To buy 8 loaves of breads, I need 100 yen more. If 30 % is discounted from the price of a loaf, 284 yen is left after buying 8 loaves. Find the price of a loaf.

Solution.

8*x* − 100 = 8*x* × (10–3)/10 + 284

*x* = 160.

(posed with partially altered)

A store sells a “tasty cookie”. A customer can buy a single cookie and a bag containing some cookies. A family of 3 persons bought 6 bags and each person ate the same number of cookies. Another family of 6 persons bought 10 bags and 10 single cookies and each person ate the same number of cookies. The numbers of cookies for one person were the same in both families. How many cookies does the bag contain?

Solution.

Let *x* denote the number of cookies in a bag.

6*x*/3 = (10*x* + 10)/6

*x* = 5.

(posed with overall altered)

##### D/D problems posed in the evaluation group

I drove from Tokyo to Nagoya. My car was driven at the speed of 100 km per hour on a highway and the journey took 4 h. Find the distance I drove.

Let *x* denote the distance I drove.

100 × 4 = *x*

*x* = 400.

(posed with overall altered)
